# Reversible Contraception in Males: An Obtainable Target?

**DOI:** 10.3390/biology13050291

**Published:** 2024-04-25

**Authors:** Joanna Nandita Koilpillai, Emily Nunan, Landon Butler, Fabio Pinaffi, Joshua T. Butcher

**Affiliations:** 1 Comparative Biomedical Sciences Graduate Program, College of Veterinary Medicine, Oklahoma State University, Stillwater, OK 74078, USA; 2 Department of Integrative Biology, Oklahoma State University, Stillwater, OK 74078, USA; 3 Department of Veterinary Medicine and Surgery, College of Veterinary Medicine, University of Missouri, Columbia, MO 65211, USA; 4 Department of Physiological Sciences, College of Veterinary Medicine, Oklahoma State University, Stillwater, OK 74078, USA

**Keywords:** male contraception, reproduction, sex hormones, nonhormonal, hormonal, veterinary, human, triptonide, One Health, comparative medicine

## Abstract

**Simple Summary:**

Historically, birth control has been geared toward targeting and subsequently reducing female fertility. The cost of unplanned pregnancies is significant, both in human and veterinary medicine. The development of an effective male contraceptive drug could provide an inclusive approach to the responsible use of contraception. The characteristics of an ideal contraceptive drug include reversibility, high efficacy, minimal adverse side effects, and easy administration. Although this seems like an uphill task, there are a few male contraceptive methods that are currently available, along with substantial interest and research being focused on novel targets. This review uses a comparative medicine approach to succinctly identify and discuss the different types of male contraceptives currently available, while also highlighting newer methods and potential targets for the future development of novel male contraceptives.

**Abstract:**

The last few decades have brought contraception to the forefront of research, with great strides made in effectively targeting and optimizing the physiology, pharmacology, and delivery processes that prevent pregnancy. However, these advances still predominantly target female contraceptives for the prevention of contraception, whereas targeting the male sex has lagged far behind. This has led to a marked deficiency in safe and effective male contraceptive agents, resulting in a heavy dependence on female contraceptives to prevent unwanted and unplanned pregnancies. Current research in the veterinary field and in rodents highlights several promising avenues whereby novel, safe, and effective male contraceptive alternatives are being developed—with an emphasis on reduced side effects and reversibility potential. This review aims to discuss current and novel male contraceptives (both human and veterinary formulations) while highlighting their efficacy, advantages, and disadvantages.

## 1. Introduction 

Control of reproduction via contraception is of great value to multiple fields, certainly to humans but also to animal breeders and for the purpose of environmental control of species (invasive or native). Unfortunately, while significant advances in contraceptive design have been made in affordability, accessibility, efficacy, and safety, the overall prevalence of contraceptive use remains heavily tilted towards the female sex. Studies regarding male methods of contraception have been ongoing for the past 50 years, similar to the work in female contraceptives; however, due to a myriad of reasons, male contraceptives have not been received with the same urgency as their female counterparts. While several reasons exist for this divide, particularly social aspects, such as the irrational fear of decreased masculinity, and thus a lack of competence, historically, female contraception has also been easier to target. As women ovulate only one oocyte per month and most males are capable of producing at least a few hundred to over a million sperm a day, targeting males would require a different approach to the existing pharmacology of female contraception. However, with the advent of better technology and understanding of the male physiology and gametogenesis across many species, formulation of a viable method of male contraception is the current goal of many researchers. Indeed, much of male contraception has been driven by the increasing need of veterinary clinicians worldwide to appropriately manage contraception in animal production and companion animals, along with certain wildlife species. This review aims to highlight some of the notable methods of male contraception, both in humans and in veterinary subjects, and to highlight potential therapeutic targets moving forward.

To date, compliance remains one of the largest barriers to contraception, especially in males. In females, a study spanning almost 30 years concluded that around 99% of women who have had sexual intercourse have used at least one contraceptive [1]. When comparing the general attitudes and willingness between males and females regarding trying a novel method of contraception, male willingness to use hypothetical contraceptives ranged from 13.6–83% [2,3], whereas female willingness in the same situation ranged from 42.8–94% [4,5]. This further demonstrates the overall social acceptance or willingness of females to utilize contraceptives compared to males. A large percentage of males use condoms or withdrawal (also known as coitus interruptus) as a method of contraception, which implies that they are willing to modulate contraception. However, preceding studies focused on these contraceptive techniques show that they are not very efficient at preventing pregnancies [6]. While condoms are a popular method of contraception, not all condoms are created equal. Preliminary studies have compared which type of condom was most effective and showed that a failure of the condom (breaking or slipping) occurred for 8.4% of couples using polyurethane condoms as their sole contraceptive and for 3.2% of those using latex condoms [6]. Another study estimated that around 60% of women between the ages of 15 and 44 years of age in the United States have used withdrawal as a form of contraception at some point in time [7]. This approach is concerning since studies have shown that live and actively motile sperm can be found in the pre-ejaculatory fluid of around 16.7% of healthy men, suggesting that merely relying on withdrawal as a method of contraception would present a high risk of contraceptive failure [8].

However, there does seem to be a shift in the prevailing mindset towards a less rigid sex-associated role in contraception. This was highlighted by a 2023 study conducted among 18- through 50-year-old cisgender men in the United States and Canada regarding participants’ willingness regarding the use of male contraception [9]. The study population was made up of 88% heterosexual males with varying familial roles in society, ranging from parents to single men, and the study noted that 75% of the respondents were willing to try novel methods of male contraception, a statistic that was strongly associated with having experienced an abortion [9]. Taken together, this would imply the theoretical social acceptance of a nonhormonal method of male contraception. This interest, if matched with focused research, could result in a breakthrough in the field of novel male contraceptive methods.

The effective development of contraceptive agents must require a One Health approach. One Health can be defined as a collaborative transdisciplinary approach to health and medicine, including human, animal, and environmental health [10,11]. One Health can often be incorrectly thought to solely focus on infectious diseases or antimicrobial resistance, but the wide scope of the One Health approach includes biodiversity management, comparative medicine, and welfare, to name other areas of focus [10]. The development of novel male contraceptives that span multiple species is a complicated and multi-step process. One of the main contributing factors to the complexity is that it requires collaboration between different subject matter specialists, including (but not limited to) medicinal chemists, pharmacologists, reproductive biologists, and clinicians to effectively create, screen, and perform clinical trials on male contraceptives [12]. 

The next generation of male contraceptives should ideally possess the following characteristics to enable a major shift in contraception demand and expectations: safe to use and administer—which must be proven by rigorous clinical trials [12]; easily available, effective, covered by medical insurance (noting that most female birth control is not covered by insurance due to the ‘elective’ nature of the drugs); and not in contradiction to the values of different social, religious, and economical groups of people, so as to increase acceptance across different groups [12,13]. Further, novel male contraceptives must largely be devoid of side effects, a key distinction in comparison to treatments of disease states, where adverse drug effects are often tolerated in lieu of increases in morbidity and mortality. 

### 1.1. Male Reproductive Physiology and Spermatogenesis

Male reproductive physiology is remarkably similar across species, especially when comparing humans with species that are within a veterinarian’s interest, with regard to intrinsic hormonal homeostasis. Some species variations do exist, such as age at puberty, type and number of secondary sex glands, type of penile tissue, site of deposition of sperm within the female tract, and seasonality of the breeding season. As examples of differences in seasonality, stallions are long-day breeders (longer periods of daylight), bucks and rams are short-day breeders (shorter periods of daylight), but bulls, dogs, and men are similarly non-seasonal breeders. However, the fundamental reproductive physiology remains the same, which is what is discussed in this section. 

**Figure 1:**
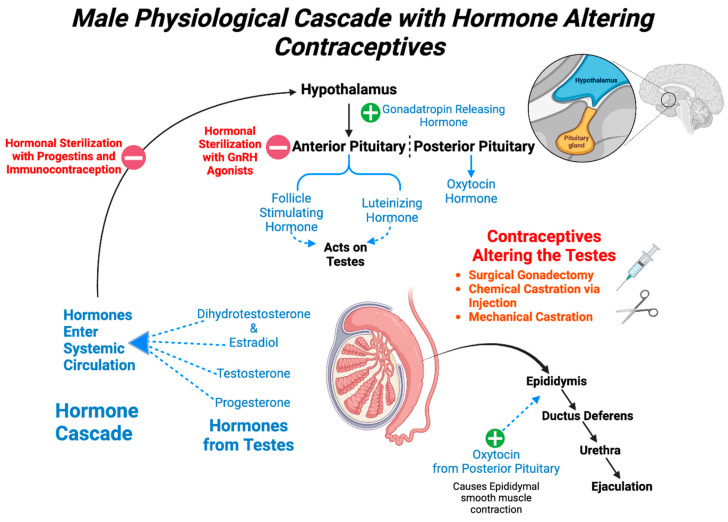
Male physiological cascade with hormone-altering contraceptives.

A brief summary of the hormones at play in male reproduction is presented below, with the purpose of highlighting the key regulatory hormones and the challenges of male contraceptive design needing to be nonhormonal to avoid systemic effects. The hypothalamic–pituitary–gonadal axis (HPG axis), as depicted in Figure 1, illustrates the complex feedback loop that aids in the self-regulation of the endogenous hormonal levels needed for male reproduction. There are four main hormones that are of clinical relevance to male contraception, namely gonadotropin-releasing hormone (GnRH), luteinizing hormone (LH), follicle-stimulating hormone (FSH), and testosterone. From the fetal stage, the hypothalamus develops differently in males and females, and this results in a different response of the anterior pituitary to GnRH stimulation, which subsequently causes LH to be secreted differently [14]. In both males and females, LH and FSH are secreted in a phasic manner with marked diurnal rhythmicity, consequent to GnRH pulsatility [14]. However, in females, in addition to this manner of secretion, there is an acute, pre-ovulatory surge of LH secretion, which causes ovulation of the mature follicle(s) [14]. As post-pubertal adults, the dynamic crosstalk between the hypothalamic–pituitary–testicular axis is tightly controlled and functionally required for overall homeostasis. The cascade begins with GnRH being released from the hypothalamus and acting on the anterior pituitary, which results in the release of LH and FSH into the circulation. In the testicle, LH acts on the Leydig cells (or interstitial cells) and FSH acts on the Sertoli cells (or sustentacular cells). The presence of both LH and FSH is required for spermatogenesis to occur. The source of testosterone is provided by the presence of LH, which induces the Leydig cells to secrete this hormone. The testosterone crosses the basement membrane and, in combination with FSH, acts on the Sertoli cells to drive sperm production in a process termed ‘spermatogenesis’. In addition to spermatogenesis, other hormones are produced by the Sertoli cells including inhibin, androgen-binding protein (ABP), and estradiol. Inhibin causes endogenous negative feedback to regulate FSH secretion. ABP helps retain the testosterone within the testicles by enhancing the selectivity of the blood–testis barrier, which ensures that the high intratesticular testosterone levels needed for spermatogenesis remain separate from the typically lower testosterone levels found within the systemic circulation. Testosterone can be converted to the highly potent androgen–dihydrotestosterone (DHT); or interconverted to estradiol by the Sertoli cells. 

Within the feedback loop, testosterone, DHT, and estradiol act as negative feedback to ensure hormonal homeostasis. It is important to note that any of the hormonal contraception used, regardless of which hormone is the active ingredient, has the potential to break the feedback loop, and result in impaired spermatogenesis. Testosterone has many positive effects on non-reproductive organs in a normal male, regardless of species. Some of these benefits include increased muscle mass, strength, and bone density, improved overall body condition, and its anabolic effect on the body [15]. As such, there is the potential for any alteration in hormone levels in males to also result in effects on endogenous testosterone production, and these effects would be felt beyond the reproductive organs. The long-term effects of using hormonal contraception are still unknown, especially with regard to its potentially altering associated hormones, and thereby altering fat, muscle, or bone mass composition [16]. This could be further complicated by additional factors such as advanced age, or obesity [17]. These factors must be taken into consideration especially when discussing contraceptives that may alter any of the physiologically reproductive hormones. 

Spermatogenesis is described as occurring in three phases: (1) the Proliferation Phase—where mitotic divisions result in a pool of immature sperm cells, which is mostly FSH driven, (2) the Meiotic Phase—where haploid, round spermatids are formed, which is driven by LH and testosterone, and (3) the Differentiation Phase or ‘spermiation’—which is the process of morphological maturation in which round, haploid spermatids transform into haploid, ‘typical sperm-shaped’ spermatozoa. Spermiation occurs primarily during transit through and storage in the epididymis, and it is at this stage that sperm cells attain basic motility prior to ejaculation. Following ejaculation, the spermatozoon undergoes its final maturation within the female reproductive tract. These steps include structural changes required to make the sperm capable of fertilizing the oocyte, such as capacitation and the acrosome reaction. Part of this process requires the sperm to maintain its motility and attain additional motility (or hypermotility); therefore, any compound that reduces sperm motility can impede the final, crucial steps of sperm transport and maturation needed for the sperm to be truly fertile. 

### 1.2. Male Contraception in Humans

In the complicated and nuanced discussion of the relative risks of contraception, men’s opinions tend to factor in different aspects when compared to women. Women typically weigh the side effects and risks of contraception against the inherent risks (i.e., health and societal) of a potential pregnancy. In contrast, men tend to prioritize their personal comfort and health risks in combination with consideration to reducing the risks to their partners. However, the shared negative effects of an unwanted pregnancy are significant and cannot be overlooked. The existing options for male human contraception fall within two categories, namely reversible (includes the usage of condoms) and irreversible (vasectomy) contraception. When used appropriately, both are highly efficient, but the reversible method is still less than 100% effective, and a vasectomy is meant to be (and often is) permanent. This presents a less-than-ideal situation wherein solely relying on reversible male contraception, at least in humans, is not practical in terms of an absolute guarantee of contraceptive success. This truly highlights the need to provide viable options for reversible contraception to choose from for males. 

## 2. Surgical (Irreversible) Contraception

Any surgical method of contraception often implies a degree of irreversibility or at minimum requires an additional surgery for reversal. Additionally, any surgery, elective or otherwise, can pose risks to a person with pre-existing conditions. Since not all people are ideal surgical candidates, this makes it harder for it to gain common acceptability as well as being economically taxing.

The most common method of surgical male contraception is undergoing a vasectomy. A vasectomy is a surgical procedure involving ligation of the vas deferens or ductus deferens. It provides contraception by occluding the passage of sperm, thereby preventing their release in the ejaculate. This surgery is typically performed as an outpatient procedure under local anesthesia by urologists, family physicians, or general surgeons in the United States [18]. It is viewed as a safe, effective, and permanent method of contraception in men. However, due to the potential irreversibility of the procedure, it can be intimidating to individuals unsure of their desire to permanently cease reproduction. The limitations of this method include extensive time management, with a need to include pre-operative medical exams to rule out any systemic and genital abnormalities, planning of the surgery, post-operative care, and recovery time. For some patients, pre-existing conditions render them poor candidates for elective surgery. Further, financial limitations must be considered. Most elective surgeries are not covered by health insurance, and the cost of the surgery (which can be roughly estimated to be around USD 1000 depending on the type of insurance coverage and where the procedure is performed) along with the loss of income from the required post-operative rest could be prohibitive. While dependent on individual patient recovery, the recommended minimum rest period would include reduced activity with intermittent cryotherapy with an ice pack for an average of 24–72 h [18]. Often, secondary loss of income can occur due to the need for a partner to be present to provide care prior to full recovery, or from surgical complications (which are very rare, but can include bleeding, hemorrhage, or surgical failure) [18]. 

Financial comparisons between existing female contraceptives reveal that routine oral birth control may be less of an immediate financial burden due to its monthly distribution, but the total cost can vary extensively (USD 0–50/month), with dependence on the insurance carrier. Within the United States, the most common emergency female contraceptive is oral levonorgestrel (Plan B) due to its over-the-counter availability, as discussed in Ref. [19], but research has shown that over the span of one year, the most cost-effective (compared against the costs of unplanned pregnancies) is actually the copper intrauterine device [19]. Additionally, a study based on the Brazilian Health System modeled cost savings from the utilization of long-acting, reversible contraceptives (LARCs) in comparison to combined oral contraceptives. It noted that all of the LARC methods were more cost-effective, despite higher upfront costs, and this becomes especially significant when considered across the span of 5 years [20]. This highlights that the cost of contraception is a present and significant factor for both female and male contraceptive agents. Thus, the incorporation of upfront costs and emergency versus long-term contraception are important considerations in comparing female and male contraceptives, and especially in the development of new male contraceptives. 

## 3. Nonsurgical Contraception

Nonsurgical methods of contraception are the most commonly used method for male contraception, mainly due to their convenience. They are often paired with another method of contraception, which can include either male or female contraception. These methods can be broadly classified into two main groups, contraceptives that do not involve the use of hormones and those that use hormones, or more succinctly, nonhormonal and hormonal contraceptives. 

## 4. Nonhormonal Contraception

The most used nonhormonal male contraceptive is the condom, which is classified as a type of barrier contraceptive. Condoms were originally made from animal intestines but were eventually composed of latex as of the 1920s. For couples with latex allergies, polyurethane condoms can be used as an alternative, but are considered less effective due to their looser fit compared to latex condoms [21]. Overall, condoms substantially reduce the spread of venereal diseases (sexually transmitted infections, or STIs) and, to a limited degree, reduce the risk of pregnancy, but condoms are not a foolproof method of contraception [22]. Condoms as the sole method of contraception are not very reliable, as the pregnancy rate for couples using condoms is 10–15% [23]. Condoms work by acting as a barrier, preventing sperm from entering the female reproductive tract and thereby preventing fertilization. 

Withdrawal is listed as the primary method of male contraception for about 3–5% of couples in the United States [24]. This approach occurs despite it not being considered a fail-proof method by medical professionals. When used as the sole method of contraception, it has a relatively high failure rate of 20–30% [23]. Withdrawal is the most common method of behavioral contraception, over abstinence. 

## 5. Male Hormonal Contraception 

Human fertility studies have shown that hormonal male contraceptives are comparable in efficacy to female contraceptives when sperm concentration is reduced to less than 3 million sperm per ejaculate [25]. Since spermatogenesis takes about 72 days in humans, treatment must be administered consistently for at least 5–8 weeks before results are effective, which is the major drawback of most hormonal preparations. Additional challenges in hormonal male contraception include the side effects of hormonal contraception; individual variation in sperm suppression based even on proven and approved drugs; long ‘on’ and ‘off’ periods, such as, if people wish to be fertile for a period of time interspersed by periods of contraception, it has a high cost, which is not affordable for many people who need contraception; and accessibility, that is, inadequate access to healthcare.

Hormonal methods of male contraception target specific hormones in the regulatory pathway of spermatogenesis and are shown schematically in Figure 1. They broadly include either a single pharmacological agent, such as an androgen (with or without testosterone), or a combination of drugs, such as combinations of progestins and androgens, gonadotropin antagonists, or androgens without testosterone. Within the HPG axis, GnRH antagonists theoretically target GnRH receptors, causing downregulation and suppression of the effect of GnRH on the cascade. Some GnRH antagonists have been studied in men paired with high doses of an androgen but there was not a significant difference between groups with and without the GnRH antagonist [26,27]. However, GnRH agonists have been successfully used as contraceptives in veterinary medicine.

Novel androgens, such as MENT (7-α-Methyl-19-nortestosterone), DMAU (dimethandrolone undecanoate), and 11-β-MNTDC (11-β-methyl-19-nortestosterone 17β-dodecylcarbonate) either bind to an estrogen receptor [28] or to both testosterone and progesterone receptors [29]. This results in a negative feedback loop to reduce GnRH secretion. A trial testing DMAU in rodents showed promising reversible effects on decreasing fertility, while still maintaining the positive extra-gonadal effects [29].

Ongoing clinical trials of a transdermal patch containing a progestin (segesterone acetate) combined with testosterone have touted it as being able to deliver a daily, self-delivered therapeutic dose of androgen, which would thereby cause contraception [30,31]. There is hope that this will be a viable alternative to the vast majority of injectable hormonal contraceptives, making them easier to administer. Testosterone blockers target androgen receptors and prevent the effect of testosterone, thus negatively affecting spermatogenesis. Flutamide is a testosterone blocker (anti-androgenic) drug along with being a nonsteroidal drug. It acts by blocking the androgen receptor from receiving signals, thereby limiting the effect of testosterone in treated animals. Theoretically, it can be used as a contraceptive as well as an adjunct therapy for testosterone-mediated neoplasms, such as prostatic cancer. However, since bioavailability has been an issue, a study was done by loading nanocarriers with flutamide versus the traditional oral or parenteral administration. The study was successful in proving that the loaded nanoparticles were able to cause a decrease in semen quality, but modifications will most likely be required based on the species of interest to formulate an appropriate carrier to allow for flutamide’s short half-life and hydrophobic nature, which would require multiple and continuous dosing to cause contraception [32].

Side effects of exogenous hormone administration include extra-testicular effects, such as pain or inflammation at the injection site, altered moods and libido, weight gain, acne, changes in cholesterol levels, and, potentially, an increased risk of cardiovascular disease [33]. Cholesterol levels are critical because all steroid hormones, mainly the reproductive hormones, are derived from cholesterol. This is compounded by the fact that spermatogenesis occurs over a period of 60–72 days [34], so this would necessitate treatment spanning more than that period to achieve therapeutic levels. This raises concerns about the safety of long-term therapy. An additional individual side effect noticed was that, in some individuals, there was a rebound in the number of sperm in ejaculate while on treatment, which could result in failure of the treatment [34]. Additionally, men from different racial backgrounds tend to respond with varying efficacy to hormonal contraception [35]. Furthermore, a lack of easily accessible and effective routes of administration, such as oral routes or transdermal patches, can be deterring to people looking for long-term treatment options, since parenteral repetitive dosing may not be a viable option for some individuals. 

The lack of focused pharmaceutical research and the costs associated with the development of male-focused contraceptive techniques coupled with the stringent quality expectations from the United States Food and Drug Administration (FDA) can also contribute to the lack of available options for male contraception [34]. A summary of human contraceptive agents is presented in Table 1, along with succinct information regarding mechanisms, hormonal alterations, and primary disadvantages.

## 6. Methods of Contraception in Veterinary Medicine

Contraception can be broadly defined as any method used to prevent the birth of offspring while maintaining fertility to potentially have offspring in the future [56]. More simply defined, contraception is the reversible control of reproduction [57]. In the field of veterinary medicine, there is a greater complexity to contraception due to species differences. While not a direct method of contraception due to its irreversibility, sterilization is often used as a primary method of controlling reproduction, resulting in the permanent cessation of reproductive cyclicity, and is mainly used in companion animal medicine. As a sequela, there is a noted decrease in reproductive behavior and physiology in both males and females [57]. As an example, neutering of stud dogs has the direct consequence of a marked reduction in sexual behavior and territorial aggression. The characteristics of an ideal permanent method of contraception in veterinary medicine largely mimic that of humans and would include safety, efficacy, requiring only a single administration, and minimal side effects with a quick recovery period if required, and an additional bonus would be if the procedure is nonsurgical, which would negate the need for equipment, anesthetic drugs, and trained personnel as well as the inherent risks of a surgical procedure [58]. 

Sterilization of companion animals, mainly of free-range, community-owned, or stray dogs or cats, has always been a need in many developing nations. One of the most significant concerns regarding this canine population includes zoonotic disease transmission to humans. For example, rabies, which is endemic in India, is a fatal, incurable, zoonotic viral disease caused by a rhabdovirus, and currently about 20,000 people die from rabies every year, with 99% of cases being caused by bites from rabid (infected) dogs [59]. 

Wildlife, including both free-ranging and captive populations, can pose unique challenges to species management [60]. Overpopulation in any form disturbs the delicate balance between species fulfilling their roles in their ecosystems to the disruption of that very balance. Historically, lethal population control methods have been commonly used to provide a correction to an overabundance of an animal population, but these do pose legal and safety concerns to both the personnel and animals involved [61]. The moral component of eliminating an animal merely for existing as an unchecked predator must also be considered. Further, challenges to successful wildlife contraception include demonstrating safety in the target species, safely and effectively administering that contraceptive agent to wildlife species with minimal stress and handling, and the ability to accurately monitor constantly changing populations [61].

Additional considerations to be taken specifically in wildlife species over domesticated species include side effects, such as changes in libido, social behavior, and group dynamics based on the hierarchy enforced by the alpha animals, potential toxicity if the drug enters the food chain by ingestion, and potential biomagnification and the ability to reverse the contraception if the population size begins to dwindle before it approaches endangered status. 

## 7. Veterinary Surgical Contraception

Historically, gonadectomy has been the contraceptive of choice and the solution to canine and feline overpopulation for decades in many countries across the world [62]. However, due to the irreversible nature of this procedure, there are an increasing number of concerns regarding the sharp decline in testosterone and activity, and subsequent increase in obesity [62]. In North America, the most prevalent method of prevention of pregnancy in small animals is surgical gonadectomy [62], with a prevalence reported of 61% in male dogs and 67% in female dogs, and 83% in tomcats and 81% in queens [57]. While the statistics presented above represent the bulk of the owned population of dogs and cats in the United States, there are still 8% that are euthanized annually at humane organizations [57]. However, this number has continually decreased over the last 20 years, and this is largely due to the effectiveness of gonadectomies [57]. As an added benefit, a gonadectomy typically takes care of unwanted sex-related behaviors and diseases associated with intact status, such as prostate issues in dogs.

Other surgical methods of sterilization include vasectomy and epididymectomy, which are less commonly performed in companion animals, but are more common in production animals to create teaser males, such as teaser bulls, bucks, or rams [63]. These teaser animals have normal serum testosterone levels, and thereby normal testosterone-related behavior, but will be unable to achieve penetration and thus, pregnancies. Post-surgical side effects of a vasectomy include chronic inflammation, formation of sperm granulomas (accumulation of sperm outside the tubules, which can rupture if sufficient pressure is applied), and less common complications, including autoimmune orchitis, scrotal and epididymal cyst formation, and fistula formation [64]. 

In farm or production animals, the need for contraception in males is comparatively lesser compared to companion animals, primarily because their main purpose is either as a replacement in the breeding stock or to be sold based on the purpose for which they are raised (ex: sustenance, wool, fiber). If not selected to be a sire, then their reproductive status is of little to no significance, and management is often easier with castrated males, such as steers (castrated bulls) and wethers (castrated bucks and rams). In the equine industry outside of show stallions, racing, or breeding operations, geldings (castrated stallions) are often preferred as pets or for non-competitive riding. Similarly, in swine production, males that are not selected for breeding (barrows) are often castrated prior to puberty.

Vasocystotomy is a surgical procedure wherein the vasa deferentia are surgically transplanted bilaterally to the urinary bladder [65]. This surgical procedure results in retrograde ejaculation, or release of sperm into the urinary bladder instead of through the penis to the external environment. Theoretically, this technique could be a surgical method of contraception that does not alter the hormonal cascade. Due to the resultant urea toxicity, osmolarity shock, and acidic pH of urine, the produced sperm would not be viable. Nikpasand et al. looked at the potential side effects of vasocystotomy in canines, monitoring semen and urine analysis six weeks after the procedure and found no reduction in serum testosterone and no post-operative complications [65]. The primary disadvantage of this procedure would be the requirement for skilled personnel, post-operative monitoring to prevent premature dehiscence of sutures (which could result in peritonitis), and a period of sexual rest until incision healing. More studies would be required to determine the actual efficacy of this procedure as a method of contraception.

## 8. Veterinary Nonsurgical Contraception

With regard to barrier contraception in dogs, records of two intrauterine devices exist, but with no significant clinical trials or efficacy trials to support them. They theoretically act by providing a barrier when placed in each uterine horn in the female [66]. Since this method is used in females, rather than in males, it is not discussed in detail in this review.

The other alternatives often include using drugs ‘off-label’ in one of three ways: firstly, the drugs are used in different species for that function without clearance for usage in the species of interest; or secondly, drugs are used in the species of interest, but for an allied purpose; or lastly, a drug is used for that purpose in the species of interest in other nations across the world, but is not cleared by the FDA for that specific use in the United States of America. In this case, the drugs in focus are not FDA-approved for use as a contraceptive agent in that species. This is often due to the high cost of obtaining FDA approval for specific usages of drugs [66].

Chemical sterilization is a nonsurgical method of contraception that typically results in inhibiting spermatogenesis due to damaged testicular parenchyma [67]. Many different compounds including glycerol, calcium chloride, dimethyl sulfoxide, and formalin have been injected bilaterally into the testicles of different species to cause irreversible sterility [67]. A comparison study compared chemical castration via a single bilateral intratesticular injection of glycerol to surgical castration in tomcats [67]. It was shown that the injected testicles underwent necrosis and fibrosis, resulting in azoospermia. The conclusions were that intratesticular injection and ligation of testicular blood supply are viable alternatives to gonadectomy and may be safer with regard to the fewer anesthetic requirements with properly managed pre- and post-operative analgesia [67]. 

An alternative method of sterilization involves destroying the intratesticular germ cells without surgically removing the testicles [57]. These sterilizing agents can be injected directly into the vas deferens, epididymis, or testicles. The main drawback of this method is that the injection causes inflammation and pain, which requires that the animal be sedated for the injection and post-treatment monitoring, which makes it less ideal from a welfare standpoint and practicality aspect for free-roaming dogs [57]. Although these techniques do not directly involve the use of a hormonal agent, the resultant destruction of the testicular architecture ends in a permanent reduction in testosterone levels, thereby disrupting the hormone profile. 

## 9. Veterinary Hormonal Contraception 

Gonadotropin-based contraceptives have been successful as the amino acid sequence of GnRH is the same in all species. Thus, translating results across species has been relatively successful [58]. GnRH agonists, such as Suprelorin, have been used ‘off-label’ in male dogs and cats for contraceptive applications [66]. Although it is not FDA-approved for use in North America, GnRH agonists are used as contraceptives in companion animals in Europe and Australia. The deslorelin implant and leuprolide acetate (slow-release injectable contraceptive) are both GnRH agonists and are the most used methods of contraception in tomcats and ferrets, and they act by disrupting the HPT axis [56]. In stud dogs and tomcats, GnRH implants effectively cause contraception since the excessive levels of GnRH cause downregulation of GnRH receptors, hence reducing LH secretion and thereby endogenous testosterone. In tomcats, the main markers of efficacy are the disappearance of penile spines and the cessation of testosterone production. A similar decease in testosterone is noticed in dogs, as well as a decrease in certain testosterone-related sexual behaviors [58]. GnRH agonists act by downregulating GnRH receptors, thereby reducing the receptivity to GnRH, thus altering the hormone cascade. GnRH agonists in the form of slow-releasing implants have been proven to be efficacious as reversible contraception in male and female dogs and cats. They can be used as adjunct therapy in pathologies exacerbated by reproductive hormones, such as prostate disease or mammary gland neoplasm [68].

Progestins or progesterone-containing drugs can be used in male dogs to reduce testosterone-related behaviors [69], but this method may not be completely effective, as some of those behaviors are learned behaviors. Similar to reducing testosterone-related behaviors in canines, progestins have been used in tomcats to prevent urine marking and other undesirable sex-linked behaviors. However, with increased progesterone levels, there is an increased risk of the incidence of weight gain, loss of coat, mammary hypertrophy, and adenocarcinoma. Additionally, progesterone and progestins can alter the binding and efficacy of testosterone and estradiol since they are common precursors in the steroid hormone cascade. Altrenogest is a progestin utilized in equine medicine to reduce stallion-like behavior, libido, total scrotal width, and the percentage of morphologically normal sperm in ejaculate, as well as serum LH and testosterone concentrations in sexually mature stallions [70]. The side effect of progestin’s use is that it must be administered at high doses for contraceptive use, and the short-term and long-term safety has yet to be determined. Exogenous progesterone administration poses serious side effects in companion animals, such as gynecomastia, mammary gland adenocarcinoma, dermatological issues, predisposition to diabetes mellitus, and suppression of the immune system [66]. Overall, hormonal methods of contraception, especially those containing progestins, have been used with increased levels of caution in male and female cats. 

## 10. Veterinary Immunocontraception 

The most well-documented attempts at immunocontraception in wildlife have centered on controlling populations in wild horses (*Equus caballus*) [71], white-tailed deer (*Odocoileus virginianus*) [72], African elephants (*Loxodonta africana*) [73], and bison (*Bison bison*) [74], amongst many other species. Immunocontraception is a method of contraception that causes the body to mount an immune response to a particular component of the fertilization pathway, thereby causing a reduction in the levels of those compounds and thereby affecting fertility. The most common targets are GnRH and the zona pellucida of the oocyte, by the GnRH and zona pellucida vaccines, respectively. The zona pellucida is a layer of the structure of the oocyte and eventually the early embryo, so it is used in females, but since GnRH is produced in both males and females, it can be used as a male contraceptive. One key reason behind the success of immunocontraception is the lack of cross-reactivity with other autologous tissues and protein hormones. This is due to effectively targeting specific targets that are essential for contraception, which allows for fewer systemic side effects. The major drawback for use in domestic species is that even though these vaccines are contraceptives, they require multiple doses, and are dependent on individual responses to the therapy and may not curb all reproductive-associated behaviors, which in companion animals are often another driving factor towards gonadectomy. 

GnRH vaccines affect sex steroids by generation of anti-GnRH antibodies, which causes a reduction in GnRH levels and subsequently a reduction in luteinizing hormone (LH) and follicle-stimulating hormone (FSH), which then also affects the production of gametes, thereby promoting contraception [75]. Immunocontraceptive vaccines can be used in both sexes within the desired species, depending on what is targeted by the vaccine. GnRH vaccines were used in free-ranging stallions but are now contraindicated, as the prevention of GnRH’s binding to its receptor prevents the GnRH stimulus for LH secretion, resulting in a lower systemic concentration of testosterone and decreased stallion-like behaviors and libido [76]. This caused a suppression in breeding behavior and can induce changes in their social groups, since wild horse populations exist as harems. Thus, for this method of contraception to be successful, every stallion in the population must be treated, or else mares may shift to another social group, and continue to foal. A similar problem arose with GnRH vaccines in mares, as they also require the majority of the mares and fillies to be vaccinated for the program to be successful. However, mares will not be as affected socially as the stallions [61]. Thus, the majority of the immunocontraceptive methods are more focused towards females of the desired species, such as porcine zona pellucida (PZP) and GnRH vaccines, which have been successfully used in multiple species, but other newer vaccines may require additional testing to prove their efficiency, both in overall contraceptive effect and also to ensure that social dynamics are not altered [75].

## 11. Veterinary Nonhormonal Contraception

There is a large gap in the availability of drugs when it comes to nonhormonal contraception in veterinary medicine. The lack of options for both males and females is likely due to a myriad of reasons, including a lack of funding or interest in obtaining FDA approval, along with how intricately complex the hormone cascade is and how involved it is in all facets of reproduction. As with human medicine, the majority of the burden of contraception has fallen to hormonal contraceptive agents used in females. However, recent research has been directed towards bridging this gap, with some promising results, which are described in the subsequent section. 

## 12. A New Frontier for Contraceptives of the Future: Experimental Nonhormonal Contraceptives 

**Figure 2:**
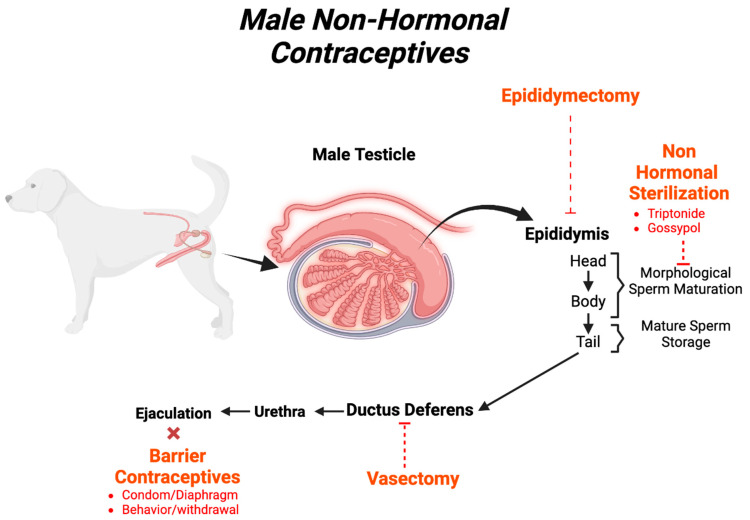
Male nonhormonal contraceptive agents.

A male nonhormonal contraceptive would be the most ideal, as it would circumvent many of the side effects accompanying hormonal contraceptives. Nonhormonal methods of contraception specifically aim to reversibly affect the testicles, prevent full maturation of sperm, or prevent a step during the production of sperm. Figure 2 highlights the mechanisms of action or locations where experimental nonhormonal contraceptives may act. Many potential male contraceptives are in different stages of being tested for efficacy and safety. This section aims to discuss some of these compounds and will conclude with a consolidated overview in Table 1 and Table 2. Promising methods include targeted cytotoxins, which can be compared to chemotherapy, in that they target and bind to specific cell types within the body. For contraceptive use, the target would be associated with reproductive cells and selectively destroy them. However, a carrier molecule must be bound to the cytotoxin to effectively deliver it to the targeted location with a high degree of accuracy, so that only the targeted cells are affected. The challenge is identifying a target only associated with reproductive function and located nowhere else in the body. Gene silencing is also quite promising but limited by similar challenges. Gene silencing uses double-stranded RNA, which when introduced to a cell will prevent transcription, and thereby expression, of a specific gene. This method requires a very high degree of precision to prevent silencing of only the desired gene and ensure that it does not provide permanent contraception. Additional research is required to understand the true efficacy of this technique. Both techniques inherently have the challenge of delivering a specific target to either the epididymis or seminal plasma to alter millions (or potentially billions) of sperm in every ejaculate, and also accounting for efficaciousness and reversibility with species variability and long periods of treatment. Despite these barriers, several promising targets, pathways, and methods have been identified and are described below. 

An additional method to occlude the vas deferens involves a surgically implantable device with a reversible valve. The device can be placed as an outpatient procedure and is able to be operated with a palpable switch. It is currently undergoing human clinical trials to determine the success of its potentially reversible nature. It is important to note that there will have to be a ‘washout’ period after closing the valve, which in theory is similar to the waiting periods after undergoing a vasectomy [38]. Reversible Inhibition of Sperm under Guidance (RISUG) is theoretically a vaso-occlusive method that is based on creating a temporary and reversible blockage to sperm flow. The blockage can be reversed by treatment with dimethyl sulfoxide (DMSO). Despite the theoretical promise of reversibility with minimal side effects, it is yet to be proven in humans, but there are initial studies showing promise in rodents and rabbits [39]. It involves injecting an occlusive styrene into the vas deferens bilaterally under ultrasound guidance, causing occlusion and thereby preventing sperm outflow [35]. This technique requires a high degree of precision and equipment to visualize the internal structures for successful placement, as well as removal, which makes this technique expensive, and it may not be widely available.

Adjudin (derived from 1H-indazole-3-caoxylic acid) has been shown to reduce sperm quality and theoretically could be considered as a nonhormonal method of contraception. It acts locally within the testicle by reducing the ability of maturing sperm to bind to the Sertoli cells, thereby causing the release of immature spermatids, resulting in a poor-quality ejaculate [41]. Two other compounds, namely gamendazole and CDB-4022, act in a similar manner to reduce fertility in rodents [45]. A study using CDB-4022 in cynomolgus monkeys showed its initial promise as a nonhormonal male contraceptive, with daily oral treatment for seven days resulting in suppression of sperm concentration and motility over six weeks, but with no changes in hormones (testosterone and LH), and with recovery by week sixteen [47]. However, clinical trials with fertility trials would be needed to prove that the contraception was truly reversible.

Gossypol is a phenolic compound obtained from the seed of the cotton plant. It is proven to reduce sperm motility and production and increase the percentage of morphologically abnormal sperm. Its mechanism of action is yet to be discovered, but fertility studies showed that it caused an approximately 90% efficacy in preventing human pregnancies. However, despite the high efficacy, around 20% of treated men did not return to pretreatment fertility levels following cessation. A concerning side effect noticed was hypokalemic periodic paralysis in around 1% of the treated men, suggestive of significant off-target systemic effects [37]. A study done in rats suggested that the contraceptive action of gossypol may be related to oxidative stress (specifically a decrease in reduced glutathione) and mitochondrial damage [78]. That study also showed that treatment with vitamin E could be used to reduce the contraceptive effects of gossypol [78]. A study in young bulls fed gossypol showed similar results, with a reduction in sperm count, sperm motility, and live and normal sperm. However, this was also accompanied by reduced libido, but all of these could be significantly improved with vitamin E supplementation [79]. Despite the promise that gossypol shows, true reversibility of contraception with fertility trials will be needed to establish gossypol as a nonhormonal male contraceptive agent.

The leaves of the small shrub plant *Justicia gendarussa*, also known as gendarussa, have been used in traditional Southeast Asian medicine for a variety of conditions, including hypertension, arthritis, and liver disease, and even as a method of male contraception. It is hypothesized to work by reversibly inhibiting the activity of the sperm enzyme, hyaluronidase, which is needed by the sperm to penetrate the oocyte, but further trials are needed to determine its efficacy as a contraceptive agent [43]. Since this contraceptive agent is yet to be proven beyond theoretical possibility, drug formulation and purification of the plant extract will be required before the contraceptive efficacy can be tested.

CatSper is a calcium ion channel specific to sperm flagella, which is essential for sperm motility [80]. Thus, blocking the CatSper channels could be a promising target for a male contraceptive by limiting sperm motility [44]. However, additional clinical trials would be needed to confirm the efficacy of the contraceptive action of CatSper. Other ion channels that are specific to sperm are being considered, such as potassium channels like SLO3, which regulates the entry of calcium through the above-mentioned CatSper channel. While the theoretical possibility of targeting ion channels would be ideal in developing a truly nonhormonal male contraceptive agent, a high degree of specificity will be required to ensure that only sperm are targeted, while the ion channels of even adjacent cells are unaffected, or else there could be major systemic side effects.

Indenopyridine derivatives prevent spermatids from binding to the seminiferous tubules [47], thereby causing them to be lost through ejaculation, and prevent mature sperm from forming. They have been tested in rodents [45], miniature stallions [46], and primates [47], which resulted in histopathological changes to the nuclei and cytoplasmic organelles of the Sertoli cells, thus reducing their functionality [46]. Reversible contraception was observed in all the above-mentioned studies, but notably, in stallions, there was no decrease in libido or sexual behaviors [46]. Despite the initial studies performed in different species, additional clinical studies with fertility trials will be required to determine contraceptive efficacy. 

Triptonide is a root derivative of the *Tripterygium wilfordii* Hook F plant, also known as ‘Lei Gong Teng’, or the ‘Vine of the Thunder God’. Historically, extracts from this plant have been used as an anti-inflammatory drug in traditional Chinese medicine [81,82]. As an incidental side effect, men who were on triptonide for a prolonged duration experienced a decrease in fertility. It has become a new focus in the search for an ideal candidate for a nonhormonal, reversible male contraceptive [13]. Indeed, triptonide is one of many derivatives from the *Tripterygium* plant [13], with others including triptolide, celastrol, demethylzeylasteral, L-epicatechin, and pristimerin. These compounds are typically separated by subfractionation followed by purification. Triptonide is a diterpenoid that is structurally very similar to triptolide, solely differing in the substituent group on the 14th carbon [83]. Both compounds have noted anti-neoplastic and anti-inflammatory properties amongst other uses; however, triptolide has proven to be metabolically more active (and also toxic) than triptonide at similar doses. One study showed that triptolide caused hepatotoxicity in mice when dosed at 1 mg/kg, whereas triptonide did not share the same negative effects on the liver at that dose [84]. Furthermore, triptolide has been demonstrated to cause testicular toxicity focused on the Sertoli cells in male mice [85], and this illustrates why triptonide is preferred over triptolide with regard to their contraceptive effects [83]. 

The exact mechanism of triptonide’s action on the male reproductive system is still not well defined. Triptonide is hypothesized to target a specific step during spermatogenesis, most likely a later step in the cascade, as the affected sperm tend to have morphological abnormalities. It is likely to be an interruption of the interaction of junction plakoglobin (JUP) with SPEM1. SPEM1 is one of a few specific genes encoding for proteins relevant solely in the late stages of developing spermatids, similar to CatSper, and encodes protein expressed only in elongating or elongated sperm [13]. Triptonide may interfere with JUP interactions with SPEM1, as treated animals’ sperm resembles SPEM null sperm, which presents with the sperm head bent backward about 180 degrees with residual cytoplasm surrounding the neck of the sperm. Treated animals rarely have a depletion of sperm within the seminiferous tubules, so the contraceptive effects likely do not involve sperm production itself. Studies focusing on the effect of oral Tripterygium on male rats also provide hints as to the potential mechanism(s) of action of triptonide on their reproductive systems. One study used testicular metabolomics and RT-qPCR to determine that the concentrations of certain metabolites, including CYP19A1, PIK3CA, and PIK3cG, changed significantly after treatment. This could be indicative of an altered glycerophospholipid, ether lipid, or glutathione metabolism in treated animals [86]. Another study suggests that Tripterygium may cause decreased cholesterol levels, which is the precursor for steroid hormone synthesis, along with a decrease in mRNA and proteins required for testosterone biosynthesis [87]. 

Clinical trials with triptonide as a contraceptive have been performed in mice and monkeys. Its reversible nature was proven along with minimal systemic side effects [13]. Histopathological analysis of the testicles revealed that testicles from the treated mice lacked elongating and elongated spermatids [13]. A reduction in fertility was proven with mating trials and was caused by a combination of oligo-astheno-teratozoospermia. Long-term testing, of up to almost 2.5 years, was carried out in primates, and no noticeable side effects were observed. The main disadvantage to this drug is that it must be given orally for about 4–6 weeks before the effect is seen in semen quality [13]. Once its safety has been established, along with its efficacy as a contraceptive agent, and with additional trials done in other species, it can be converted to either a depot injection or a slow-release capsule without the requirement of daily dosing. There are many plants with derivative compounds that could potentially be used as contraceptives; however, triptonide is claimed to be one of the most widely studied plant-based methods for male contraception. 

Genomic-based studies could theoretically be useful in determining whether alterations to specific sperm proteins could result in potentially reversible, nonhormonal male contraception. The identification of novel targets should be stratified by the impact on sperm quantity and quality, and further differentiated to include decreased total sperm numbers (oligozoospermia), an increased percentage of morphologically abnormal sperm (teratozoospermia), and the decreased motility of sperm, both total and progressive motility (asthenozoospermia). Methods of contraception that cause asthenozoospermia or reduced sperm motility often act at the stage of sperm hypermotility during the capacitation cascade since the hypermotility of sperm at this step is directly proportional to their fertilizing ability. These methods must target substances that affect the haploid, post-ejaculation spermatozoon and ensure a reduction in fertility to reliably cause contraception [88]. 

The bromodomain testis-specific protein (BRDT) is a testis-specific protein that is required for meiosis, and for that reason is a good target to inhibit potential contraception. It has been noted that individuals with a defect in the BRDT gene experience reduced fertility due to morphological abnormalities—specifically, abnormally shaped sperm heads, which reduces the quality of the ejaculate, thereby decreasing fertility [48]. It has been shown that JQ1 is a small molecule that reversibly inhibits BRDT function in mice [39]. The downside is that JQ1 nonselectively inhibits other members of the bromodomain family, so the challenge is to identify a drug that does not cause toxicity at the therapeutic contraceptive dose of BRDT inhibition. 

During spermiation, one of the crucial steps involves a testis-specific serine kinase (TSSK) protein family. TSSK is expressed in mature sperm after they completely undergo meiosis [89]. The TSSK family of proteins is highly conserved and present in many different species, which could potentially make them good candidates for a male nonhormonal contraceptive [89]. Additional considerations should be taken for those males who are already oligospermic or asthenospermic, as they may be affected differently from seemingly healthy and fertile males. 

Morphologically mature sperm are stored in the tail of the epididymis prior to ejaculation. Post-ejaculation, they are exposed to a bicarbonate-rich (more alkaline) environment coupled with the activation of soluble adenylyl cyclase (sAC). This leads to morphological changes, which are crucial for fertilization. Thus, targeting sAC could theoretically act as a nonhormonal contraceptive agent. The proof of concept has been accomplished in mice, with the deletion of the sAC genes being dose-responsive, as heterozygous deletion reduces sAC activity and homozygous deletion results in the total abolition of sAC activity [90]. Recently, this concept has been extended, as targeting sAC via the use of an oral sAC inhibitor was proven to be an effective and temporary contraceptive in male mice [40]. 

Epididymal peptidase inhibitor (‘EPPIN’) is a protein located on the surface of sperm cells. It is theoretically a good target for male contraception as it is expressed solely in the male reproductive tract, specifically the testes and epididymides [91]. A study worked on an organic compound, EP055, that targets EPPIN on the spermatozoa and reduces its mobility [42]. It was proven to be effective at decreasing sperm quality in male cynomolgus monkeys [42]. However, clinical and fertility trials will be required to determine the contraceptive efficacy.

Vitamin A and its associated metabolite, retinoic acid, are necessary for the initiation of spermatogenesis at puberty and the continued sperm production throughout the adult reproductive life [49]. Retinoic acid binds to one of the numerous retinoic acid receptors (RAR). Studies have shown that male RAR knockout mice are sterile [50,51]. An oral compound consisting of a retinoic acid receptor antagonist has been formulated that inhibits three types of retinoic acid receptors. Initial trials resulted in testicular degeneration as well as liver inflammation in rats. However, in mice, reversible contraception was demonstrated [52]. Another potential method of nonhormonal contraception of merit includes inhibiting retinoic acid biosynthesis. WIN 18,446 resulted in contraception by inhibiting testicular retinoic acid, and briefly underwent clinical trials in men [55]. However, when the treated men consumed alcohol, they experienced severe side effects, such as palpitations, nausea, and sweating, so the trial ended early. Subsequently, the mechanism of action was understood to be inhibition of testicular retinoic acid biosynthesis by inhibiting certain dehydrogenase enzymes [53,54]. So, current research is seeking to formulate a novel compound that can inhibit testicular retinoic acid without affecting alcohol consumption [92]. A summary of veterinary contraceptive agents is presented in Table 2, along with succinct information regarding mechanisms, hormonal alterations, and primary disadvantages.

## 13. Conclusions

Contraception is a vital component in a successful One Health paradigm, represented by the integrated and unified approach between people, animals, and ecosystems due to their closely linked and interdependent health (World Health Organization), and is especially important in society today. Indeed, the human race is not beyond notice, as there are significant consequences to an unsustainable birth rate. Currently, fertility rates are well below the replacement level, which suggests that contraceptives are less a population control issue and more a matter of individual choice [93]. In human medicine, contraception remains a right, which is exercised “based on full information and without coercion” [94], as quoted by the American Nurses Association’s position statement on reproductive health. However, in veterinary medicine, contraception is key to ensuring that a particular species is facing neither overpopulation (as in the case of invasive species) nor threats to extinction (an increasing threat commonly observed in wildlife). 

The concept of reversibility is key in encouraging responsible usage of contraception, both in human and veterinary medicine. Permanent sterility carries inherent risks for any species, as the balance between overpopulation and extinction is likely more delicate than can currently be modeled. A general reduction in species biodiversity across the globe is being driven by climate change and the subsequent (potentially irreversible) alteration of habitats, along with the greater ease with which invasive species can be introduced. In 2019, a study based on the Endangered Species Act (ESA) in the United States identified a total of 97 ESA-listed species that were extinct [95]. The ESA-listed species could typically be sighted prior to their extinction for a mere 2–23 years [95]. 

The historical track record whereby the female sex has borne the brunt of contraceptive responsibility leaves only one tool in the proverbial contraceptive belt. As such, the development of a viable male contraceptive would enable a more equitable approach to contraception and allow for a complete One Health paradigm. The ideal target for a male contraceptive will likely need to be a nonhormonal method of contraception that specifically affects sperm production or maturation and is independent of major systemic side effects (with both short- and long-term use). 

While often overlooked, veterinary medicine currently stands at the forefront of this field due to the varied complexities observed within theriogenology, including effective management of domestic and production animals, controlling invasive species, and preventing the decline of exotic wildlife. Significant advances will likely be driven by needs within this circle. With human use, additional factors also need to be considered, such as an understanding of male compliance, differing levels of social/cultural acceptance, and potential barriers or biases that can also negatively affect the response to male contraception. Future concepts, such as fair pricing policies and health care coverage, would aid in improving acceptance for contraception in any form, especially if it is a novel method being introduced. Furthermore, accessible public sex education, with a focus on educating people of all ages regarding the safe usage of contraceptives, may help with destigmatizing the use of contraceptives, especially male contraceptive agents. 

Extending the variety of types of contraception will be more inclusive for those individuals and their partners (both female and male) who may not be suitable candidates for traditional birth control. The same is true of contraception in veterinary medicine. A varied, multi-pronged approach to contraception in both human and veterinary medicine will ultimately decrease the financial burden being allocated to terminating unwanted pregnancies and the medical costs associated with those pregnancies. As the current trend in medicine is shifting towards a One Health approach, clinical contraception should also be changing, and developing an effective, reversible, nonhormonal male contraceptive agent would be a key step in that direction. 

## Figures and Tables

**Table 1:** Male contraceptive agents in human medicine.

No.	Human Male Contraceptives	Mechanism of Contraception	Hormone Status Alteration	Contraceptive Efficacy	Main Disadvantage
1	Vasectomy	Occlusive contraception by bilaterally ligating the vas deferens	-	Proven > 98% efficiency with an appropriate washout period [36]	Surgical risks
2	Condoms	Barrier contraception—no sperm in ejaculate	-	Only 70% effective if sole contraceptive method [23]	Efficacy is less than ideal [6,21]
3	Nal-Glu	GnRH antagonists—reduced endogenous GnRH production	Yes	Approx. 90% efficacy when combined with an androgen [27]	No proven efficacy if used as the sole contraceptive [26]
4	MENT, DMAU, 11ß-MNTDC	Androgen-based reduced endogenous androgen production	Yes	Yet to be proven beyond pilot studies	Extra-gonadal hormonal side effects [29]
5	Segesterone acetate	Progestin-based altered hormone cascade	Yes	Yet to be proven beyond initial clinical trials	No proven evidence of efficacy
6	Flutamide	Testosterone blocker—blocking testosterone receptors from binding with endogenous testosterone	Yes	Initial studies show promise, but carrier molecule must be further developed [32]	Low oral bioavailability [32]
7	Gossypol	Reduced semen quality—exact mechanism is yet to be discovered **	-	90% efficacy in human fertility trials [37]	Hypokalemic periodic paralysis [37]
8	Vas deferens occlusive device	Occlusive contraception by a reversible valve in an implantable device	-	Undergoing human clinical trials [38,39]	No proven evidence of efficacy
9	Soluble adenylyl cyclase (sAC) targeting	sAC is required to morphologically mature sperm between epididymal storage and ejaculation [40]	-	Yet to be proven beyond theoretical possibility [40]	No proven evidence of efficacy
10	Adjudin, gamendazole, CDB-4022	Reduced binding ability of maturing sperm to Sertoli cells [41]	-	Yet to be proven beyond theoretical possibility [41]	No proven evidence of efficacy
11	Epididymal peptidase inhibitor (EPPIN)	Targeting a surface protein located on sperm; only expressed in the male reproductive tract	-	Pilot study showed promise for short-term contraception in cynomolgus monkeys with no listed efficacy [42]	Yet to be proven effective in fertility trials
12	Gendarussa leaves	Potentially reversing sperm hyaluronidase activity [43] **	-	Yet to be proven beyond theoretical possibility	No proven evidence of efficacy
13	Calcium ion channel in sperm flagellum (CatSper)	Blocking the ion channel—reducing the motility of the sperm [44]	-	Yet to be proven beyond theoretical possibility	No proven evidence of efficacy
14	Indenopyridine derivatives	Preventing spermatids from binding to the seminiferous tubules	-	Studies done in rodents [45], miniature stallions [46], and primates [47] but no contraceptive efficacy listed	Initial studies show reversible contraception, but variability in libido and sexual behaviors
15	JQ1 bromodomain testis-specific protein (BRDT) inhibitor	Required for meiosis—causing morphologically abnormal sperm—specifically abnormally shaped sperm heads [48]	-	Pilot study done in mice showed promise, but no contraceptive efficacy listed [48]	Nonselective BRDT inhibition—multiple side effects and higher doses required for contraception
16	Vitamin A and metabolite retinoic acid	Binds to one of many retinoic acid receptors (RARs) [49]. RAR mice are noted to be sterile [50,51].	-	Pilot study done in mice showed promise, but no contraceptive efficacy listed [52]	Liver inflammation seen in rats [52]
17	WIN 18,446	Inhibiting retinoic acid biosynthesis by inhibiting certain dehydrogenase enzymes [53,54]	-	Clinical trial in men showed initial promise, but no contraceptive efficacy listed [55]	Severe side effects when combined with alcohol consumption—palpitations and nausea, etc. [55]

** Mechanism of action not yet fully understood despite an apparent reduction in male fertility.

**Table 2:** Male contraceptive agents in veterinary medicine.

No.	Veterinary Male Contraceptives	Mechanism of Contraception	Hormone Status Alteration	Contraceptive Efficacy	Main Disadvantage
1	Gonadectomy/castration—surgical and with mechanical castrators	Permanent, bilateral removal of the testicles [62,77]	Yes	Proven 100% effective in all species [62]	Irreversible contraception with disruption to the hormone cascade [62]
2	Vasectomy	Occlusive contraception by bilaterally ligating the vas deferens	-	Proven 100% effective with appropriate washout period [63]	Irreversible contraception [63]
3	Epididymectomy	Occlusive contraception by bilaterally ligating the epididymis	-	Proven nearly 100% effective [63] with appropriate washout period	Irreversible contraception [63]
4	Vasocystotomy	Surgical redirection of the vas deferens, resulting in retrograde ejaculation into the urinary bladder [65]	-	Yet to be proven beyond pilot studies	Surgical risk and requirement of skilled personnel
5	Chemical castration with glycerol, calcium chloride, DMSO, formalin	Damages testicular parenchyma, which reduces spermatogenesis and testosterone production [67]	Yes	Can theoretically be used in any species [67]. Contraceptive efficacy varies by chemical.	Testicular necrosis and fibrosis with associated pain
6	GnRH agonists: deslorelin, leuprolide	Downregulation of receptor’s sensitivity to endogenous GnRH	Yes	Proven effective in many companion animals [58]. Contraceptive efficacy depends on the formulation.	Off-label use in most species due to limited formulations
7	Progestins like altrenogest	Progestin-based altered hormone cascade [69]	Yes	Proven effective in dogs [69], stallions [70], but contraceptive efficacy varies by formulation	Numerous side effects due to increased progesterone levels
8	Immunocontraception—GnRH vaccines	Causes an immune response to the GnRH molecule, thereby reducing endogenous production of GnRH	Yes	Proven effective in multiple domestic and wild species [71,72,73,74]. Contraceptive efficacy varies by species and vaccine formulation.	Not an ideal contraceptive in all species based on population dynamics [75,76]
9	Gene silencing	Prevents expression of critical genes by inhibiting their transcription **	-	Yet to be proven beyond theoretical possibility	Very high degree of precision required for formulation
10	Testis-specific serine kinase (TSSK) protein targeting	Potential target since it is essential for sperm maturation and conserved among species	-	Yet to be proven beyond theoretical possibility	No proven evidence of efficacy
11	Reversible sperm inhibition—RISUG	Occlusive contraception temporarily blocks the release of sperm. Reversed with DMSO.	-	Yet to be proven beyond pilot studies	Yet to be proven effective in fertility trials
12	Triptonide	Altered spermatogenesis resulting in morphologically abnormal sperm—not fully understood **	-	Proven effective in mice and monkeys with 100% penetrance [13]	Only available as an oral formulation requiring daily dosing

** Mechanism of action not yet fully understood despite an apparent reduction in male fertility.
